# Evaluation of Home Health Care Devices: Remote Usability Assessment

**DOI:** 10.2196/humanfactors.4570

**Published:** 2015-06-05

**Authors:** Philip Kortum, S Camille Peres

**Affiliations:** ^1^ Rice University Department of Psychology Houston, TX United States; ^2^ Texas A&M University Health Science Center Department of Environmental and Occupational Health College Station, TX United States

**Keywords:** health care evaluation mechanisms, human-computer interaction design and evaluation methods, patient satisfaction, usability testing

## Abstract

**Background:**

An increasing amount of health care is now performed in a home setting, away from the hospital. While there is growing anecdotal evidence about the difficulty patients and caregivers have using increasingly complex health care devices in the home, there has been little systematic scientific study to quantify the global nature of home health care device usability in the field. Research has tended to focus on a handful of devices, making it difficult to gain a broad view of the usability of home-care devices in general.

**Objective:**

The objective of this paper is to describe a remote usability assessment method using the System Usability Scale (SUS), and to report on the usability of a broad range of health care devices using this metric.

**Methods:**

A total of 271 participants selected and rated up to 10 home health care devices of their choice using the SUS, which scores usability from 0 (unusable) to 100 (highly usable). Participants rated a total of 455 devices in their own home without an experimenter present.

**Results:**

Usability scores ranged from 98 (oxygen masks) to 59 (home hormone test kits). An analysis conducted on devices that had at least 10 ratings showed that the effect of device on SUS scores was significant (*P*<.001), and that the usability of these devices was on the low end when compared with other commonly used items in the home, such as microwave ovens and telephones.

**Conclusions:**

A large database of usability scores for home health care devices collected using this remote methodology would be beneficial for physicians, patients, and their caregivers.

## Introduction

### Overview

The usability of technology can be important in the consumer domain because it can drive adoption and create consumer loyalty [1]. In the medical domain, however, lack of usability can cost lives. In 2000, the Institute of Medicine (IOM) published its seminal report, *To Err is Human: Building a Safer Health System*, indicating that over 98,000 lives were lost every year in the United States alone due to preventable human errors [2]. Recent reports suggest that this number may have grown to over 400,000 [3]. Although the IOM report focused on the errors that were occurring in hospitals, they noted that “…as more care shifts to ambulatory and home settings, the use of medical technology by non-health professionals can be expected to take on increasing importance” [2] (p 63). Indeed, if some of the most highly trained medical professionals in the world are making errors in the treatment of patients, there should be great concern in the ability of patients and their care providers to render medical treatment at home without error.

Challenges for home care are rising for multiple reasons. First, an increasing amount of health care is now done in a home setting, away from the hospital. From 1995 to 2008, there was a fivefold increase in the number of patients who received home health care from Medicaid, with an estimated 12 million people receiving some form of home health care [4]. Second, this increase in home health care is being accompanied by ever increasing levels of technology being used in the home. Third, the individuals who are expected to use this technology are likely to be minimally trained, working under stressful conditions, and may be suffering from age-related declines in cognitive, perceptual, and physical abilities—circumstances that can lead to the potential for errors, often with significant consequences [5].

The United States Food and Drug Administration (FDA) has also recognized that home health care devices are of growing importance and concern with the launching of their Medical Device Home Use Initiative in 2010 [6]. As part of that initiative, the organization has acknowledged that there are many benefits of using health care devices in the home, including cost savings resulting from fewer hospitalization days and the potential for improvements in the quality of life certain patients may enjoy because they are in a familiar and convenient venue as they receive their care. However, they also note that there are numerous usability issues surrounding the use of such devices and that these issues need to be systematically addressed.

Often times the difficulty in home-care device use stems from the fact that devices that have been designed and certified for professional medical users are then directly transferred to the home environment with little regard to the difficulty this might pose. These kinds of transitions from hospital use to patient/caregiver use might be more successful if it were acknowledged that these 2 user populations are different, and have different needs, and then these differences could be accounted for in the design process or during the development of training material. An excellent case in point is the migration of defibrillator technology from the sole domain of trained medical professional use to use by a completely untrained general population. In a study of hospital-grade defibrillators, experienced emergency medical services personnel made errors (such as trying to defibrillate before the device was ready and performing a cardioversion when they intended to defibrillate), which could cause harm to the patient [7]. This would seem to suggest that migration of this kind of mission-critical device to public use would be ill advised. However, after significant user-centered design work on the development of automatic external defibrillators (AEDs), studies have shown that untrained 6th grade school children’s performance with the device was comparable with that of professional paramedics [8]. This success suggests that with proper care, even complex medical devices can be made safe and effective for use by relatively untrained individuals.

### Background

For some time now, the anecdotal evidence about the difficulty of ease of use for home health care devices has been building. However, there has been little systematic scientific study to quantify the global nature of the home health care device usability problem and characterize device usability in a field situation. Much of the available literature has tended to focus around a handful of devices, such as pregnancy test kits [9], cholesterol test kits [10], glucometers, and other diabetes management tools [11]. Studies typically assess a few medical devices of a single type in a laboratory setting, making it difficult to compare usability across studies and devices. It also makes the pace of adding new usability information about specific devices exceptionally slow.

There is a growing consensus that the usability of home-care devices warrants significant additional attention. The Association for the Advancement of Medical Instrumentation (AAMI) recently released standards [12] that address the human factors requirements for highly usable medical devices and the US FDA has begun to enforce the application of these standards in the approval process of new devices. Numerous groups, including AAMI, the US FDA, the National Academies of Science, and the Human Factors and Ergonomics Society have held numerous forums, panels, and workshops aimed directly at the human factors issues associated with home health care, with the goal of highlighting the importance of the problem and to disseminate the latest research findings.

One of the key pieces of information that is currently lacking in this domain is a quantitative assessment of the usability of a broad range of home health care devices. Designers, physicians, home health nurses, caregivers, and patients would all benefit by having a better understanding of how usable (or unusable) different home health care technologies really are. Physicians could use the information to make more informed decisions about what kinds of home health care might be appropriate for their patient, particularly those who might have physical or cognitive declines. Home health care nurses could use the information to determine what devices might need extra attention when showing a household member how to use that device. Patients and family caregivers could use the information to help select home health care devices that had the best usability profiles. Further, patient compliance and adherence to medical advice is a known issue [13] and patients and caregivers are much more likely to adopt and use medical devices if they believe that those devices will be easy to use [14].

Indeed, poor home health care device usability made it to the ECRI Institute’s top 10 health technology hazards of 2012 [15]. One of their recommendations was for doctors to consider the usability of the devices they were going to prescribe for their patients. However, this information does not currently exist for the wide variety of home health care devices currently being used.

There are a number of ways that this kind of usability data could be collected. Traditional user testing is one important way. Traditional user testing takes place in a laboratory and involves bringing in representative users, giving them a task to perform, and observing their performance as they try to accomplish the task on the given product or service. The International Standards Organization usability metrics [16] of effectiveness (accuracy and completion of tasks), efficiency (time on task, physical or mental effort, rate of throughput), and satisfaction are generally collected and used to assess the usability of the product. This kind of testing could also take place in a home or hospital setting or it could take place remotely, with the experimenter conducting the test from a distant location, while the patient uses a device in the home. Other evaluation methods, often described as discount-usability techniques, could also be employed. In these methods, experts make assessments of the product or service (without benefit of real users) by employing a set of usability heuristics and determining how well the product conforms to those heuristics. The difficulty with these traditional methods is that they require extensive time to perform, and so the number of devices that can be evaluated, as well as the number of users who can evaluate each device, is greatly limited.

In this paper, we describe a very different method of collecting data—a remote usability assessment method using a survey that captures a user’s assessment of the usability of a product or service, or in this case, a home health care device. The advantage of using such a method is that it allows for a much broader and larger sample, and eliminates some of the issues associated with small usability samples [17]. More users from diverse groups can be assessed and a greater number of devices can be evaluated than with traditional methods. More importantly, users can base their usability assessments on the totality of all their experiences with the device, rather than a single in-laboratory interaction. The method can be applied to a specific brand and model of device (eg, Acme Glucometer Model X-123) or to a class of devices, without regard to a specific model or brand (eg, glucometers). Collecting data on classes of products allows researchers to make more generalizable assessments of products that might have usability difficulties due to the nature of the task they perform, or the technology required to perform that task. While there are undoubtedly differences in the usability of specific products within a class, it has been shown that the variance of the usability scores for classes of items is the same as the variance observed for usability scores of a specific item [18]. This suggests that there is general agreement about the average usability of a class of items. For example, it seems likely that most readers would agree that a standard touch-tone landline telephone is easier to use than a handheld global positioning system navigation system. Indeed, Kortum and Bangor [18] used this remote method to collect data on 14 different popular consumer goods (for both specific items and classes of items) for over 1000 users and found that the method produced reliable data. Further, Kortum and Peres [19] found that this method is comparable with usability testing for ordinally comparing the usability of devices or systems.

## Methods

### Data Collection

Usability data on home health care devices were collected in the field remotely, without direct usability testing. Using the System Usability Scale (SUS), participants were asked to rate the subjective usability of common home health care devices with which they had direct experience.

### Participants

The participants were 271 undergraduate students at Rice University (Houston, TX, USA). There were 161 female participants, 109 male participants, and 1 who responded as “other” to gender, with an average age of 19.5. Participants self-selected into the study, and were not recruited or screened on the basis of having any specific health issues.

### Measures

In this study, we used the SUS to assess subjective usability. The SUS is a 10-item survey instrument developed by Brooke [20] as an efficient method of determining the usability of a given product or service. There are a large number of other surveys available that also measure usability (see [21] for a review), but the SUS was chosen because it has 5 attributes that make it ideal for use in this study. First, the survey has demonstrated that it can be used to assess nearly any technology, so any number of different devices or interfaces can be assessed with the same instrument [21]. While many of these evaluated technologies have been consumer-grade systems, the SUS has also been used successfully in the medical domain for devices as diverse as insulin pumps, heart rate monitors, and glucose-monitoring devices. Second, the SUS has high reliability and has been used in a large number of studies, and therefore, its properties are well-known, with well-established benchmarks for comparative analysis [21-23]. Third, because of its short length, it can be quickly and easily administered. Fourth, the survey returns a scored value between 0 (unusable) and 100 (highly usable), which makes the interpretation of the scores easier for experts and nonexperts alike. Research relating these scores to easily understandable adjective ratings has made the interpretation of the scores even easier [24]. Finally, because the instrument is nonproprietary, it is a cost-effective choice for researchers to use.

In this study, we used the modified version of the SUS described by Bangor and colleagues [21]. This version differs from the original version of the SUS with a simple modification of question 8 (changing the word “cumbersome” to “awkward”) to increase its understandability for a broader range of raters. The SUS was further modified by changing the word “system” to “medical device” to assist the user in making accurate ratings. This type of change has been demonstrated to have no impact on the validity or reliability of the survey instrument [23].

### Procedure

Upon signing up for the study, participants were directed to a website that contained the survey. After completing an Institutional Review Board-approved consent form, they were queried about basic demographic information and given general instructions that described the rating task and provided exemplars of the kinds of home health care devices that were of interest. They then selected a home health care device that they had used from a list (Table 1), which was a subset of home health care devices described by Story [25]. Because our sample population comprised relatively healthy students, we only used 23 of the devices listed by Story [25], excluding those devices associated with more acute care (eg, nasogastric feeding tubes, hospital beds). Following this selection, they rated that device’s usability using the SUS. There was also an option for the participant to enter any other home health care device they used, and rate its usability as well. The participant continued rating until they reached 10 devices or indicated they had no more devices to rate.

## Results

### Devices Rated

The participants rated a total of 455 devices. Table 1 shows the specific devices rated by the participants and the frequency of the ratings, as well as the mean and standard error of the SUS scores. As seen in Table 1, the thermometer was rated by the most participants and had one of the higher average SUS scores (80.53). The highest SUS score was for the oxygen mask (95.00), but this was only rated by 2 people. There were 8 different devices that participants listed under “other” and those were stethoscope, nebulizer, allergy nasal spray, humidifier, electronic muscle stimulator, electroencephalography (EEG), intrauterine birth control device, and BAND-AID.

**Table 1:** Frequency of responses for each device, as well as the mean and standard error (standard error of the mean) of the System Usability Scale (SUS) for each device.

Device	N	Mean SUS^a^	Standard error of the mean
Thermometer	227	80.53	0.88
Blood pressure cuff	71	73.56	1.72
Inhaler	62	75.97	2.15
Pregnancy test kit	23	66.74	4.27
Syringe	16	67.66	4.58
Blood glucose meter	12	69.58	5.34
Epinephrine injector (EpiPen)	11	65.00	4.89
Allergy test kit	7	71.43	4.81
Drug test kit	4	68.75	11.97
Feeding tubes	2	71.25	6.25
Hormone test kit	2	58.75	16.25
Nebulizer	2	67.50	12.50
Oxygen masks	2	95.00	5.00
BAND-AID	1	—	—
Birth control: intrauterine device	1	—	—
Catheters	1	—	—
Cholesterol test kit	1	—	—
Electroencephalography (EEG)	1	—	—
Electrocardiogram monitors	1	—	—
Electronic muscle stimulator	1	—	—
Humidifier	1	—	—
Intravenous equipment	1	—	—
Nasal spray (allergy)	1	—	—
Stethoscope	1	—	—
Transcutaneous electrical nerve stimulation equipment	1	—	—
Ventilators	1	—	—

^a^The SUS scores are not reported for those devices that had only 1 response.

### Differences Between Devices

To determine whether there were any reliable differences between devices in their subjective usability, a one-way analysis of variance was conducted with the SUS as the dependent variable and medical device as the independent variable. This analysis was only done for those devices that had more than 10 responses, which included the following: thermometer, blood pressure cuff, inhaler, pregnancy test kit, syringe, blood glucose meter, and epinephrine injector (EpiPen).

**Figure 1:**
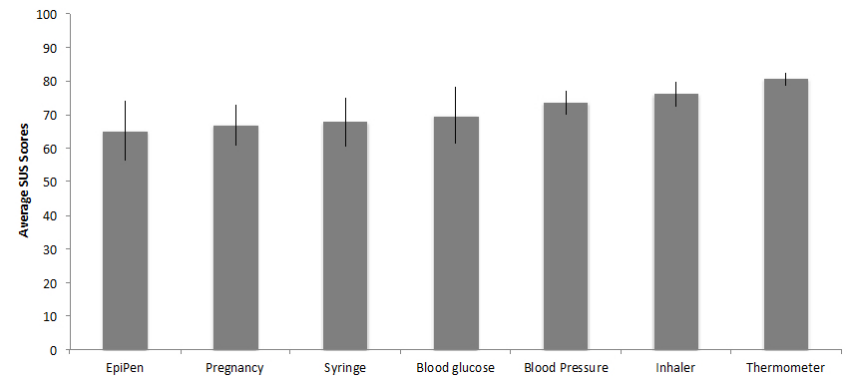
Mean System Usability Scale scores by device. Error bars represent the 95% CI.

Figure 1 shows the average SUS scores by devices for those devices that had more than 10 responses. As seen in this figure, the thermometer had the highest score and the EpiPen had the lowest. The effect of device on SUS scores was significant, *F*_6,413_=7.27, *P*<.001, η^2^=.096, and a Tukey post hoc analysis found that the thermometer usability was significantly higher than the blood pressure cuff (*P*=.014), EpiPen (*P*=.018), pregnancy test kit (*P*=.001), and syringe (*P*=.02).

## Discussion

### Principal Findings

In this study, we examined the usability of a number of home health care devices using a field-based retrospective methodology and the SUS. The study was designed to determine the usability characteristics of these medical devices in realistic settings, and set the stage for much larger data-collection efforts using this method in the future, to much more fully characterize the usability of home health care devices. This study yielded several important findings. First, even with this young, healthy, and well-educated sample population, a wide variety of home health care devices was used. Second, the usability of these rated devices covered a fairly wide range, and third, there were statistically significant differences (see the “Results” section) in the subjective usability ratings given to these different devices. These findings suggest that this method could be used to great effect to more fully characterize a broader range of home health care devices.

Because medical devices in the home are often used in critical life and death situations and can also be an important part of maintaining a healthy life, it is essential to understand how usable these home health care devices are. If a user cannot successfully use their complex new television remote control, then the result is simply an inability to watch television. If a user fails to successfully and correctly use a home health care device, the impact could be significantly greater, up to and including death. This study used a convenience sample of young, well-educated users to make these evaluations. From the results obtained, it is reasonable to be concerned that people who are ill and using more complex devices will have similar or (likely) worse experiences with their home health devices.

**Figure 2:**
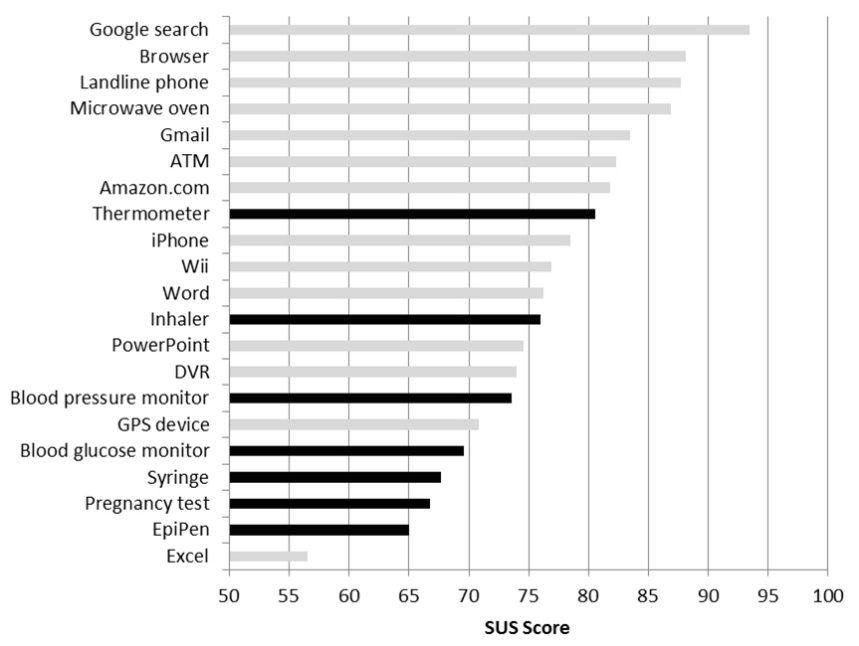
Comparison of usability ratings for the home health care devices in this study (black bars) and 14 common products described by Kortum and Bangor [18] (gray bars).

Many home health care devices are, in large part, another consumer item. They are widely available to the general public and are sold in both traditional brick-and-mortar retail outlets and through general merchandize online outlets such as Amazon.com, Inc. Because many of these devices are no longer the sole purview of specialized medical device retailers, it seems likely that consumers may view these devices as another commodity and will make assessments of the usability and utility of home health care devices in the same way that they make assessments of other consumer goods. The question of how the usability of these home health care devices compares to other common devices used by the general public is instructive because one would expect (and hope) that home health care devices would have higher ease of use characteristics, given the importance of their function. Figure 2 shows how these home health care devices compare to 14 other kinds of commonly used software programs and devices that were described by Kortum and Bangor [18]. Remarkably, the rated medical devices are some of the most unusable. As can be seen, they occupy 5 of the 7 lowest scores, when compared with these common devices used by the general public. Only the inhaler and the thermometer score in the middle of the pack. Of particular note is the rating given to the EpiPen. This is a device that must be used correctly, at a time and place not of the user’s choosing, under conditions that can only be described as exceedingly stressful. Failure to use the device correctly within a very small time frame can result in death. There is no time to consult the instruction manual and no time to call for technical support. And yet, even with this mission criticality, the device was rated very low in its subjective usability.

**Figure 3:**
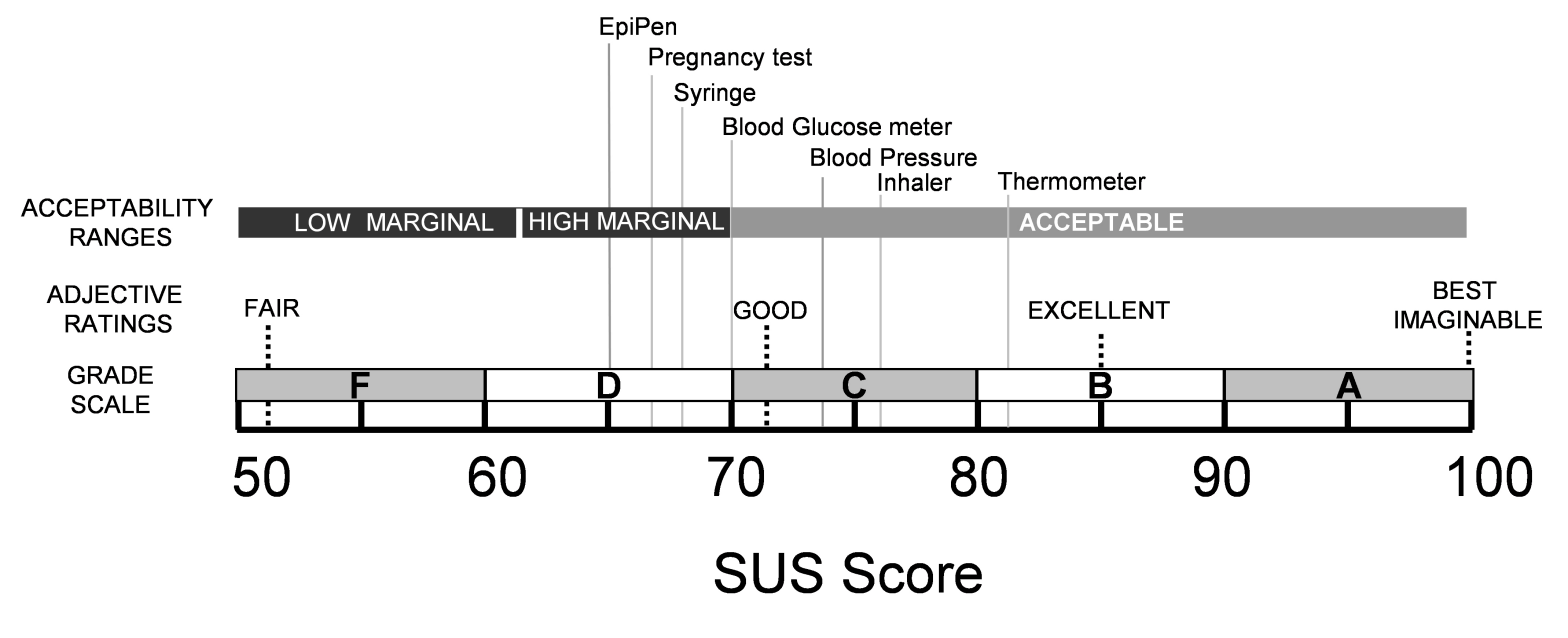
Usability ratings of the home health care devices plotted on the acceptability scale [24]. Scores below 50 are judged to be unacceptable.

If we plot these devices on the Usability Acceptability Scale [24], it can be seen (Figure 3) that over half of the rated devices are in the “marginal” range, with the remaining ones being judged as “acceptable.” Clearly, no medical device should be in the “marginal” or “unacceptable” ranges, particularly those that have life-or-death consequences.

### Future Directions

The data show that there is wide variability in the usability of different home health devices, even among a relatively young, healthy population of users. Further research needs to be undertaken to explore how usability ratings may differ by demographic variables such as health status, age, socioeconomic status, and education levels. With this expansion in demographics, the classes of devices that users can rate will also need to be expanded to accommodate home health devices that are used more in chronic care (eg, dialysis machines, lift equipment). This expanded data would also allow for the construction of more relevant comparisons with the usability of other home health care devices, rather than just with common consumer devices, as shown in Figure 2. This would more accurately reflect the frequency (eg, regular use of an inhaler and infrequent use of an EpiPen) and nature of the interactions (eg, critical versus noncritical) that occur with home health care devices in the field. Further research should also be conducted to determine whether the adjective rating scales found with general consumer products (Figure 3) are still appropriate for home health care devices.

This kind of future research would set the stage for communicating with physicians, hospitals, and patients about the specific kinds of home health care devices which have sufficiently poor usability that their use, as is typically prescribed, might represent a risk to the health of the patients using them. From that information, further work can be undertaken to determine what could be done to mitigate or eliminate these risks.

These steps might include working directly with manufacturers and physicians to identify methods of directly providing information about the need for increased patient contact and training when certain classes of devices are prescribed. For example, it could be that when physicians prescribe the use of a home health care device, a “usability risk database” is referenced, which alerts the physician that he or she will need to follow-up more frequently with patients using these devices. Manufacturers could communicate with the users of devices through their instruction books or warning labels to alert users of low-usability/high-risk devices that “this piece of equipment must involve extensive training before use in a home health environment.” In the longer term, a dissemination mechanism, such as a website, could be constructed such that consumer groups, physicians, manufacturers, patients, and caregivers could search for usability information for specific types of home health care devices (akin to Consumer Reports). This website could be linked to major sources of medical information such as WebMD or Wikipedia, making it readily and easily available when patients and caregivers are making health decisions.

All of these dissemination mechanisms would have the sole goal of educating critical personnel in the care chain (physicians, nurses, patients, and patient caregivers) about the usability of a wide range of home health care devices. Consumers have ready access to this kind of information for all manners of other consumer goods, but there is a gap when it comes to many home medical devices. The method of remotely collecting usability data described in this paper would allow for the creation of these kinds of medical device usability information databases. These databases would, in turn, provide valuable information on the usability of devices throughout their life cycle.

Although there are many benefits to be derived from a larger scale collection of subjective usability data for home health care devices, interpretation of the data must be done carefully. The correlational relationship between task performance and subjective usability assessment is not perfect [23]. There may be cases where devices have acceptable task performance in the field, but are judged poorly with subjective usability measures. In this case, the need for further attention to the device would be captured and the benefits of additional design work could be measured against the time and cost of modifying a device that has otherwise sufficient performance properties. More concerning would be those devices that receive acceptable-to-high subjective usability scores, but have poor performance characteristics. In this case, the need for further attention to these devices might not be noted, because performance data are not specifically captured in this remote protocol. Further research should be conducted to determine the relationship between subjective and objective usability measures for home health care devices and if there are methods to accurately capture device performance elements from the questionnaire format.

### Conclusions

Understanding the usability of home health care devices is important as more health care is pushed into the home. Patients, who used to be cared for primarily in hospitals or long-term care facilities, are now routinely sent home with a myriad of medical devices to manage and treat their conditions. With a sufficiently expanded data-collection effort, the kind of usability data described here could be used to impact not only the design and development of future devices, but also could be used immediately to help physicians and patients alike make better, more informed decisions when prescribing or choosing home health care equipment.

As always, the more information a physician and patient can share about the patient’s care, the better that care can be. The continuing transition from hospital care to home health care means that the usability of devices now used in the home needs to more fully understood, and this information needs to be shared, so that care can be delivered in the safest, most effective possible way.

## Abbreviations

AAMI: Association for the Advancement of Medical Instrumentation
AED: automatic external defibrillator
EEG: electroencephalography
FDA: United States Food and Drug Administration
IOM: Institute of Medicine
SUS: System Usability Scale
